# 

**Published:** 1912-12-21

**Authors:** 


### The Pharmaceutical Society Benevolent Fund.

### THE SUCCESSFUL ANNUITANTS.

An election in connection with the Benevolent Fund of the Pharmaceutical Society was held at 17 Bloomsbury Square, London, W.C., last week, when eight candidates appeared for four annuities. The names of those elected are as follows: Mr. H. G. Collins (aged 72), £50 per annum; Mr. R. Dawson (aged 73), £50 per annum; Mrs. F. H. Litteljohn (aged 59), £40 per annum; Mrs. J. A. Pimm (aged 60), £40 per annum. The highest and lowest number of votes polled were 4,994 and 3,047 respectively.

It is noteworthy that the benefits of the fund are not confined to members of the Society, but "to all persons who have been registered as pharmaceutical chemists or chemists and druggists, whether connected with the Society or not, and for the widows of such persons." For the year ending December 1911 there were no less than thirty-one annuitants participating in its benefits.

The attention of all institutional pharmacists is particularly drawn to the fact that in addition to the annuities and casual relief of the Benevolent Fund, there is also attached to it an Orphan Fund, whereby children of deceased pharmacists who have been members of the Society and have contributed to the Benevolent Fund for a period of three years (if only 2s. 6d. per annum) are also taken in hand by the Society.

### Buildings Contemplated.

Edinburgh.—New tuberculosis dispensary buildings have been completed at a cost of £8,326 from plans by Messrs. Mitchell and Wilson. They include administrative and educational departments. On one side are the dispensary, two research laboratories, and an *x*-ray room. A board room, museum, and lecture hall are also provided.

Glasgow.—An additional storey is projected at Glasgow Royal Infirmary. Mr. J. Millar, A.R.S.A., is the architect, and tenders are invited.

Hinckley.—The Local Government Board has fixed a date for an inquiry in connection with sanction to a loan for an infectious diseases hospital to serve Hinckley district.

Kettering.—Plans have been prepared by Messrs. Blackwell and Riddey for extensions to the Kettering Infectious Hospital. The Local Government Board has held an inquiry for sanction to a £1,400 loan.

### Gifts and Bequests.

Mrs. Anne Wright Tate, of Ryde, has left £1,000 to the Royal Isle of Wight County Hospital.

Mr. Austen Chamberlain has received £200 from the Suez Canal Company for the fund which he is raising to extend the London School of Tropical Medicine.

Mrs. Eliza Smith, of Brighton, has left £3,000 to the Sussex County Hospital; £1,000 to the Alexandra Hospital for Children, for founding the "David Smith Cot or Cots"; and £1,000 to the London Hospital.

Mrs. Mary Ann Batchellor, of South Kensington, has bequeathed £6,000 to King Edward's Hospital Fund for London, £500 to the London Hospital, Whitechapel, and £1,000 on trust for one life, with remainder to the London Hospital.

### AN IRISH SURGEON'S BEQUEST.

Mr. Robert John Montgomery, F.R.C.S.I., M.A., M.B., of Dublin, has left personal estate valued at £4,823. He has bequeathed £5,000 to the Board of Dublin University and to the Royal College of Surgeons, Ireland, for a "Mary Louisa Montgomery Lectureship" in Ophthalmology, to be held alternately by the boards for a period of five years, the lectureship for the first five years after his death being held by Dublin University. The testator added that should the income of his sisters, apart from that from real estate, amount to less than £200 per annum, then it shall be made up to that amount from the said fund.

